# COVID-19 Vaccines in Cancer Patients. Seropositivity and Safety. Systematic Review and Meta-Analysis

**DOI:** 10.3390/vaccines9091048

**Published:** 2021-09-20

**Authors:** Luigi Cavanna, Chiara Citterio, Ilaria Toscani

**Affiliations:** Oncology and Hematology Department, Piacenza General Hospital, Via Taverna 49, 29121 Piacenza, Italy; c.citterio@ausl.pc.it (C.C.); i.toscani@ausl.pc.it (I.T.)

**Keywords:** cancer, hematologic malignances, COVID-19, vaccines, antibodies

## Abstract

Patients with cancer are among the most vulnerable groups of the COVID-19 pandemic, whereas vaccinations can represent a cornerstone in overcoming the pandemic itself. However, cancer patients were excluded from clinical trials for COVID-19 vaccinations, and thus the data on the immunogenicity and safety of COVID-19 vaccines in cancer patients are limited. In this systematic review, we assessed the seroconversion rate and the safety of COVID-19 vaccinations in cancer patients. We searched a bibliographic database up until 31 July 2021. Utilizing inclusion criteria, six studies were selected and analyzed for this meta-analysis. This included 621 cancer patients and 256 controls. Results show that patients with solid tumors show adequate antibody responses (>90%), though the antibody titers were significantly lower than those of healthy controls. Similarly, a significantly lower rate of seroconversion was registered in patients with hematologic malignances. The vaccines showed a good safety profile; no grade 3–4 adverse events were registered. This review demonstrates generally high immunogenicity from COVID-19 vaccines in patients with cancer, with better results for solid tumors than hematological malignances, and with a good safety profile.

## 1. Introduction

Coronavirus disease 2019 (COVID-19) is a highly infectious virus that has caused significant discomfort and death worldwide. It has been reported that mortality from COVID-19 is higher among patients with cancer [1,2,3]. Most cancer patients are elderly and have other comorbidities, such as hypertension, diabetes, coronary disease, and chronic obstructive pulmonary disease, which are risk factors for severe disease and death [2,3]. Cancer patients are at high risk of acquiring COVID-19 due to poor general conditions and a deficient systemic immunosuppressive state caused by cancer and/or by anticancer treatment. In addition, cancer patients have frequent scheduled visits to hospitals and clinics, which can increase their risk of catching COVID-19 [4]. As previously reported, patients with cancer have a markedly elevated risk of intubation, intensive care unit (ICU) admission, and death, whether these patients are receiving active anticancer treatment or are cancer survivors [5].

We have previously reported cases involving the first 25 cancer patients with COVID-19 pneumonia in the Western world and discovered a mortality rate of 36.00% [2]. Additionally, cases involving 51 cancer patients with COVID-19 were reported upon by our group, and we found a COVID-19-related mortality rate of 23.53% [3]. Given the greater severity of COVID-19 in cancer patients and their higher risks of death, these patients are considered to be a high-priority subgroup for vaccination against COVID-19, and while vaccines against COVID-19 have shown a high efficacy, immunocompromised patients were not included in controlled trials [6]. Limited data have been available regarding the efficacy, tolerability, and safety of COVID-19 vaccines in patients with cancer, as cancer patients were excluded from clinical trials of COVID-19 vaccines [7]. It must be emphasized that the major organizations of Western countries, such as the American Society of Clinical Oncology, the Association of Cancer Research, and the Association of American Cancer Institutes in the United States, as well as the European Society of Medical Oncology, the Society of Immunotherapy and Cancer, and the Italian Medical Oncology Association in Europe, have recommended the vaccination of all cancer patients, including those receiving active anticancer therapy [8,9,10,11,12,13,14]. COVID-19 vaccines were approved and recommended by the US Food and Drug Administration and the European Medicines Agency in order to prevent the COVID-19 disease. In phase 3 trials, the majority of these vaccines showed 94% to 95% efficacy in preventing symptomatic SARS-CoV-2 infection independent of age [15,16]. Patients with cancer clearly represent a highly susceptible group that needs to be protected by effective and safe vaccines [17]; however, there is a paucity of data on the efficacy and safety of available vaccines against COVID-19 for cancer patients [18,19,20,21,22]. For these reasons, the examination of COVID-19 vaccinations’ potential effectiveness and safety among cancer patients may be of particular importance. The aim of this review was to assess efficacy of COVID-19 vaccinations among cancer patients, evaluated by seroconversion, as well as to assess the safety for the same group.

## 2. Materials and Methods

The study was designed according to the standards set forth by a relevant statement from PRISMA (Preferred Reporting Items for Systematic Reviews and Meta-Analyses) [23]. The obtained online data included papers published in English in PubMed, Cochrane Library, and medRxiv up until the date of 31 Jul 2021. The following search words were used: (COVID [Title] OR COVID-19 [Title] OR coronavirus [Title] OR SARS-CoV-2 [Title] OR 2019-nCoV [Title]) AND (vaccine [Title] OR vaccination [Title] OR Seroconversion [Title] OR Seropositivity [Title]) AND (cancer [Title] OR oncologic [Title] OR hematologic [Title]). The research was limited to full text results.

### 2.1. Study Selection

We included observational prospective, retrospective, and cross sectional studies. The inclusion criteria were age ≥ 18 years old, cancer patients (solid tumor or hematologic malignancies), vaccination for SARS-CoV-2, seropositivity evaluation, and presence of a control population. Editorials, letters, reviews, case reports, case series, and commentaries were excluded.

Two investigators (L.C., C.C.) independently extracted the following information: first author, vaccine used, study design, sex, mean age, number of oncologic patients, number of control, and seroconversion rate. Disagreements regarding the data were resolved by consultation with a third author (I.T.). To identify additional studies, the bibliographic references were verified, and publications considered relevant were searched for manually. We examined asymmetry in funnel plots with standard errors plotted against effect size and Egger’s tests of significance to assess potential publication bias.

### 2.2. Statistical Analysis

RStudio 3.6.0 software was used for the statistical analysis. Fixed or random effects were applied as part of an analysis model depending on the heterogeneity across studies; we used the Mantel–Haenszel method. The heterogeneity was estimated using the I^2^ statistic. Effects were presented as a risk ratio (RR), with a corresponding 95% confidence interval (CI).

## 3. Results

**Figure 1:**
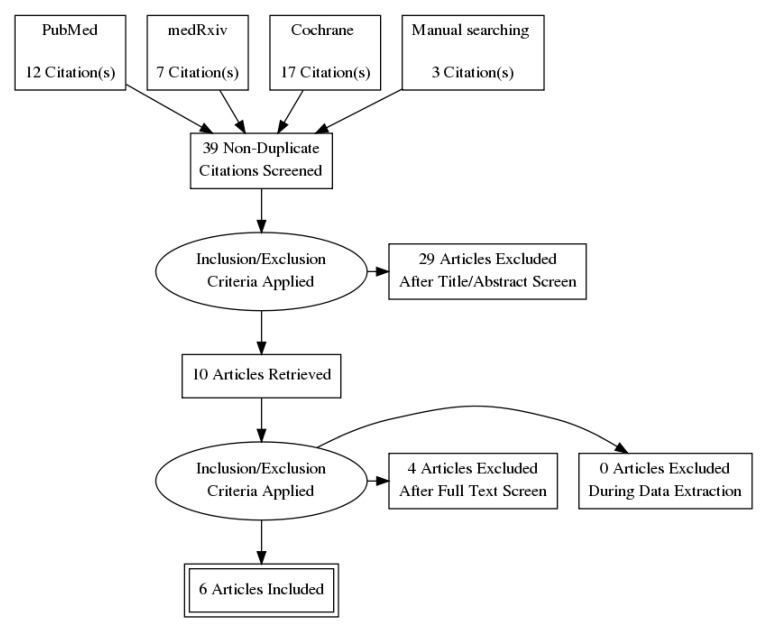
PRISMA diagram.

By searching the online database according to the search strategy, 12 articles from PubMed, 17 from the Cochrane Library, and 7 from medRxiv were initially identified. Further manual searching via the use of reference lists from pertinent articles was carried out, and another three studies were added. According to the inclusion and exclusion criteria, six studies (two cross-sectional and four prospective case-controlled studies) were ultimately included for the final analyses (Figure 1).

Three of these studies had performed serological tests using the anti-SARS-CoV-2 spike protein antibody test (the Abbott method was employed for this test) [17,18,20], whereas another had used the ELISA test [19], another the lecsys Anti-SARS-CoV-2 S assay [22], whereas the final had used the LIAISON^®^ SARS-CoV-2 S1/S2 IgG test [21]. RT-PCR was used in three studies as a reference point for the diagnosis of SARS-CoV-2 infection [19,20,21]. Four studies utilized a double-injection BNT162b2 vaccine [18,20,21,22]; each of the patients were evaluated for anti-SARS-CoV-2 IgG responses. In one study [17], 115 patients (57.5%) had received the BNT162b2 vaccine, and 62 (31%) had received the mRNA-1273 mRNA vaccine. Another 20 (10%) had received the single dose of Ad26.COV2.S23, and 3 patients (1.5%) had received a complete vaccination, but data regarding the serologic responses were not available. In the second study [19] a total of 31/151 (20.53%) patients and 16/54 (29.63%) controls had received their first doses of the BNT162b2 vaccine only, whereas 31/151 (20.53%) of patients and 16/54 (29.63%) controls had received 21-day vaccine boosters. Only 24/31 (77.42%) patients and 12/16 (75%) controls were evaluated for anti-SARS-CoV-2 IgG responses. The vaccines performed were two doses of the mRNA vaccine BNT162b2 [15], administered 21 days apart [17,18,19,20,21,22]; or two doses of the mRNA-1273 vaccine [16] 28 days apart [17]; or else simply one dose [24] of the adenoviral vaccine Ad26.COV2.S [17].

A total of 621 cancer patients and 256 controls were included in the meta-analysis. The baseline characteristics of the included studies are summarized in Table 1. 

There were 621 cancer patients and 256 controls. A total of 281 patients with solid tumors and 340 with hematologic malignances were evaluated. Overall, 173 patients were not receiving active anticancer treatment during vaccination. The patients’ median age ranged from 66 to 82 years (67 [17], 82 [18], 73 [19], 66 [20], 70/73 [21], 71 [22]).

All studies included in the meta-analysis reported upon the rate of seropositivity following COVID-19 vaccination as primary outcomes. Five studies [18,20,21,22] reported comparisons of SARS-CoV-2 IgG titers between cancer patients and healthy controls (Table 2). 

Three authors [17,19,21] performed a safety analysis (Table 3) and reported that vaccinations appeared to be generally very safe, with mostly mild and moderate adverse effects reported. Monin et al. [19] also reported that vaccinations were well tolerated. Pimpinelli et al. [21] reported that only two patients reported severe pain after their second doses, though no serious adverse event was registered. Iacono et al. [18] and Herishanu et al. [22] did not perform safety analyses, but the former reported no major adverse events related to the vaccination, and the latter reported only mild and moderate adverse events.

**Figure 2:**
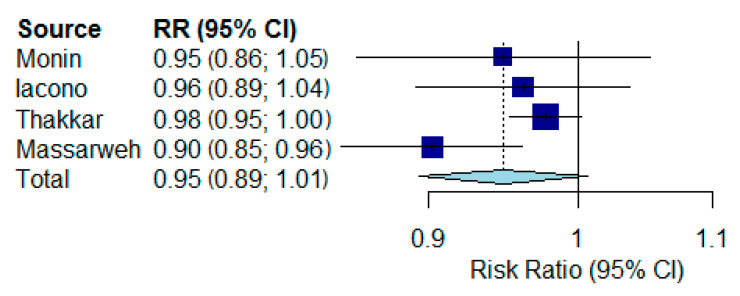
Forest plots of seroconversion rates comparing vaccinated patients with solid tumors with the vaccinated control. RR: risk ratio; CI: confidence interval.

For the meta-analysis, we separated the oncologic and hematologic patients. Considering that heterogeneity was significant, a random effects model was used for the analysis. The results of the first meta-analysis are reported in Figure 2; we found no reduced rate of seroconversion for vaccinated oncologic patients with solid tumors compared with the control (RR 0.95, 95% CI 0.90 to 1.01, *p* = 0.09, I2 = 73.5%, *p* = 0.01).

**Figure 3:**
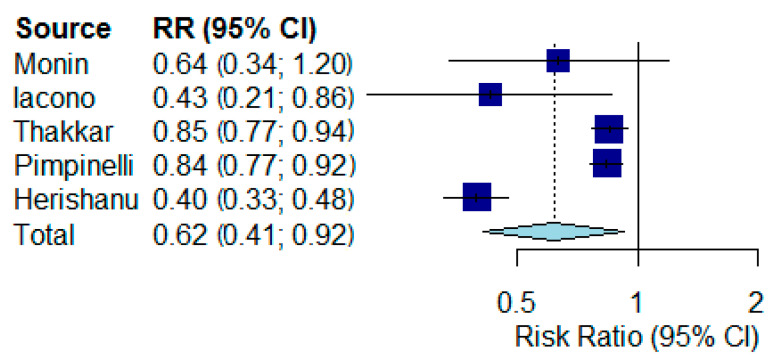
Forest plots of seroconversion rates comparing vaccinated patients with hematological malignances versus vaccinated controls. RR: risk ratio; CI: confidence interval.

For the hematologic patient (Figure 3), the difference was significant. There was a reduced rate of seroconversion for vaccinated hematologic patients compared with controls (RR 0.62, 95% CI 0.41 to 0.92, *p* = 0.02, I2 = 96.2%, *p* < 0.001). For the meta-analysis, high degrees of heterogeneity were shown.

Asymmetric funnel plots were produced; both indicated a possibility of publication bias, though the Egger’s tests were not significant (*t* = −1.24, *p* = 0.34 for oncologic patients and *t* = −1.13, *p* = 0.34 for hematologic patients). We did not detect clear publication bias, as the number of included studies was small

## 4. Discussion

The complexity of care of cancer patients—including treatments such as surgery, chemotherapy, target therapy, immunotherapy, and radiation therapy—poses unique challenges at the time of the COVID-19 pandemic. Oncologists, in addition to cancer patients, must ultimately be protected from unnecessary exposure [25]. It has been reported from China that about 1% of patients infected with COVID-19 had cancer, which is at a rate of 5 times higher than the general incidence rate in China [5]. In addition, a report from Italy evidenced that about 20% of the deceased patients with COVID-19 had cancer within the past five years [26].

Patients with cancer were excluded from pivotal clinical trials for COVID-19 vaccines, despite being included in the priority category for COVID-19 vulnerability [27], and there is a paucity of data on the efficacy and safety of mRNA COVID-19 vaccines in cancer patients so we believe that our study will be useful. To the best of our knowledge, this is the first comprehensive meta-analysis regarding the immunogenicity and safety of anti-SARS-CoV-2 vaccines for cancer patients. In the present meta-analysis, we showed that the majority of patients with cancer had immunogenic responses to COVID-19 vaccinations. In the study of Thakkar et al. [17], a higher seroconversion rate was reported in patients with solid tumors (98%), compared with a lower rate of seroconversion (85%) observed in patients with hematologic malignances, particularly in patients following highly immunosuppressive therapies such as anti-CD20 monoclonal antibodies treatment (79%) and stem cell transplantations (73%). The adverse effect profiles of each type of vaccine in this study are considered to be acceptable. In the analysis reported by Monin et al. [19], which enrolled both patients and health care workers who had received the mRNA BNT162b2 vaccine after three weeks, 97% of health care workers had immune responses (anti-S IgG positive titers) with a single inoculation, whereas only 39% of patients with solid tumors and 13% of patients with hematological malignances experienced seroconversion with single inoculums. It must be emphasized that patients with poor seroconversion after single vaccine injections were patients who had received chemotherapy in a time period of less than 15 days since receiving the vaccine or were patients with thoracic malignances. On the other hand, after a second dose of the vaccine, 95% of patients with solid tumors showed seroconversion. No grade 3–4 adverse events were reported. Iacono et al. [18] similarly reported upon serologic responses to COVID-19 vaccinations in older patients (≥80 years) with cancer. Among these 36 frail patients, 10 suffered from hematologic malignances and 26 from solid tumors. The seroconversion rates were 40% and 96.75%, respectively, without grade 3–4 adverse events. Massarweth et al. [20] evaluated rates of anti-spike (anti-S) antibody responses to the BNT162b2 vaccine in patients with cancer (solid tumor) who were undergoing systematic treatment (102 patients), along with those of 78 healthy controls. The seropositivity rates following two doses of the vaccine were 90% in the patients group and 100% in the control group, respectively. No grade 3–4 adverse events were reported. Herishanu et al. [22] evaluated the humoral immune responses to the BNT162b2 mRNA COVID-19 vaccine in 167 patients with chronic lymphocytic leukemia (CLL); the initial antibody response rate was found to be low: 39.5%. However, the response rate was better for the patients with CLL who obtained clinical remission after treatment (79.2%). This was followed by a rate of 55.2% for treatment-naïve patients and of only 16% among patients undergoing active treatment at the time of vaccination. Finally, a 0% rate was found for patients who had been exposed to anti-CD20 antibodies in a time period of 12 months before vaccination. Pimpinelli et al. [21] reported upon the immunogenicity and safety of anti-SARS-CoV-2 BNT162b2 vaccines for patients with hematologic malignances. They analyzed 42 patients with multiple myeloma (MM) and 50 with myeloproliferative malignances (MPM). Seroconversion rate was at a rate of 78.6% for MM patients, 88% for MPM patients—and at 100% for the control group. No safety concerns were observed. The results of the studies included in this meta-analysis were comparable and coherent with each other. Some limitations were found to be present in this meta-analysis. First of all, the limited number of patients in each study impacts the final statistical power. Moreover, the limited observation period of the patients does not allow for a response to the pertinent question: How long does the antibody protection last? In addition, the antibody levels alone, in the absence of concomitant investigations on T cell responses, do not allow for a complete assessment of immunity in response to COVID-19 vaccinations [28].

## 5. Conclusions

In this meta-analysis of 281 unselected cancer patients with solid tumors, the anti-S antibody response rate following the complete planned inoculation of a COVID-19 vaccine was found to be promising (>90%). The antibody response to a COVID-19 vaccine, was, as expected, inferior for the 340 patients with hematologic malignances. Nonetheless this meta-analysis suggests that COVID-19 vaccination for cancer patients is both effective and safe and that it should be prioritized. Patients with cancer who have already been vaccinated, however, should continue wearing masks, and practicing social distancing and hand hygiene practices.

## Figures and Tables

**Table 1:** Baseline characteristics of completed COVID-19 vaccinations (2 doses of BNT162b2, 2 doses of mRNA-1273, or 1 dose of Ad26.COV2.S) in the included studies (Ad: Ad26.COV2.S; BNT: BNT162b2; f: female; CS: cross sectional; MM: multiple myeloma; MPM: myeloproliferative malignancy; mRNA: mRNA-1273; nr: not recorded; PR: prospective; Sc: seroconverted).

Author Reference /Country	Vaccine/ *n*. Inoculations	Study Design	Tot. Patients	Sex (f%) of Patients/ Controls	Age Median (Range) Patients/ Controls	Patients on Active Treatment *n*(%)	Solid Tumors		Hematologic Malignances		Controls	
							Tot	Sc *n*(%)	Tot	Sc *n*(%)	Tot	Sc *n*(%)
Thakkar et al. [17]/USA	BNT/2, mRNA/2, Ad */1	CS	200	58/62	67 (27–90)/ 64 (37–82)	135 (67.5)	134	131 (97.76)	66	56 (84.85)	26	26 (100)
Iacono et al. [18]/Italy	BNT/2	CS	36	58.4/nr	82/nr ^§^	31 (86)	26	25 (96.15)	10	4 (40)	72	52 (100)
Monin et al. [19]/UK	BNT/2	PR	24	48/48 ^#^	73 (64.5–79.5)/ 40.5 (31.3–50) ^#^	13 (45.83)	19	18 (94.74)	5	3 (60)	12	12 (100)
Massarweh et al. [20]/ Israel	BNT/2	PR	102	43/68	66 (56–72)/ 62 (49–70)	102 (100)	102	92 (90.2)	0	0	78	78 (100)
Pimpinelli et al. [21]/Italy	BNT/2	PR	92	MM 45.23, MPM 48/50	MM 73 (47–78) MPM 70 (28–80)/ 81 (79–87)	92 (100)	0	0	MM 42 MPM 50	33 (78.6) 44 (88)	36	36 (100)
Herishanuet al. [22]/Israel	BNT/2	PR	167	32.9	71 (63–76)/ 68 (64–74)	75 (44.9)	0	0	167	66 (39.52)	52	52 (100)

* Ad26.COV2.S single dose vaccine used in 20 patients, ^§^ aged ≥ 66, ^#^ data relating to the entire study population, blood samples 2 weeks following a 21-day vaccine available only for 24 patients.

**Table 2:** Serum IgG level pre- and post-vaccination for cancer patients and controls (CI: 95% confidence interval; EC_50_: half maximal effective concentration; GMC: geometric mean concentration; IQR: interquartile range MM: multiple myeloma; MPM: myeloproliferative malignancy; np: not performed; nr: not reported).

Author Reference	Positive Cutoff/Unit	Median Serum IgG Level (Pre-Vaccinations)	Patients’ Median Serum IgG Level (Post-Vaccinations)	Controls’ Median Serum IgG Level (Post-Vaccinations)
Thakkar et al. [17]	≥50 AU/mL	np	Solid tumors 7858 Hematologic malignancies 2528	Higher than 15,000
Iacono et al. [18]	≥50 AU/mL	np	2396.10 (range 0–32,763)	8737.49 (range 398.90–976,280)
Monin et al. [19]	70 EC_50_ dilution units	np	nr	nr
Massarweh et al. [20]	≥50 AU/mL	np	1931 (IQR, 509–4386)	7160 (IQR, 3129–11,241)
Pimpinelli et al. [21]	≥15 AU/mL	GMC (CI): MM 4.2 (3.9–4.6) MPM 4.6 (4.2–5.2) Controls 3.8 (3.8–3.8)	GMC (CI): MM (42 patients) 106.7 (62.3–179.7). MPM (50 patients) 172.9 (106.5–257.0)	GMC (CI): 353.3 (255.6–470.0)
Herishanu et al. [22]	≥0.80 U/mL	np	0.824 (IQR, 0.4–167.3)	1084 (IQR, 128.9–1879)

**Table 3:** Adverse events after second vaccine doses. G: grade (grade 1 (mild; does not interfere with activity); grade 2 (moderate; interferes with activity), grade 3 (severe; prevents daily activity), and grade 4 (potentially life-threatening; emergency department visit or admission to hospital)); AE: adverse event.

Author Reference	AE G1–2 *n*(%)	AE G3–4 *n*(%)
Thakkar et al. [17]	BNT152b2 37% mRNA1273 34% Ad26.COV2 26%	0
Iacono et al. [18]	Not reported	0
Monin et al. [19]	29% patients, 69% controls	0
Massarweh et al. [20]	Not reported	Not reported
Pimpinelli et al. [21]	Most common side effect 16% pain	0
Herishanu et al. [22]	39 (23.4)	0

## Data Availability

The datasets generated during and/or analyzed during the current study are available from the corresponding author on reasonable request.

## References

[1] Chamilos G., Lionakis M.S., Kontoyiannis D.P. (2021). Are all patients with cancer at heightened risk for severe Coronavirus Disease 2019 (COVID-19)?. Clin. Infect. Dis..

[2] Stroppa E.M., Toscani I., Citterio C., Anselmi E., Zaffignani E., Codeluppi M., Cavanna L. (2020). Coronavirus Disease-2019 in cancer patients. A report of the first 25 cancer patients in a Western country (Italy). Futur. Oncol..

[3] Cavanna L., Citterio C., Toscani I., Franco C., Magnacavallo A., Caprioli S., Cattadori E., Di Nunzio C., Pane R., Schiavo R. (2020). Cancer patients with COVID-19: A retrospective study of 51 patients in the district of Piacenza, Northern Italy. Futur. Sci. OA.

[4] Bitar N., Kattan J., Kourie H.R., Mukherji D., El Saghir N. Caring for Cancer Patients during the COVID-19 Pandemic. http://www.lsmo-lb.org/news/caring-for-cancer-patients-during-the-covid-19-pandemic.

[5] Liang W., Guan W., Chen R., Wang W., Li J., Xu K., Li C., Ai Q., Lu W., Liang H. (2020). Cancer patients in SARS-CoV-2 infection: A nationwide analysis in China. Lancet Oncol..

[6] Shroff R.T., Chalasani P., Wei R., Pennington D., Quirk G., Schoenle M.V., Uhrlaub J.L., Ripperger T.J., Jergović M., Dalgai S. (2021). Immune responses to COVID-19 mRNA vaccines in patients with solid tumors on active, immunosuppressive cancer therapy. medRxiv.

[7] Desai A., Gainor J.F., Hegde A., Schram A.M., Curigliano G., Pal S., Liu S.V., Halmos B., Groisberg R., Grande E. (2021). COVID-19 vaccine guidance for patients with cancer participating in oncology clinical trials. Nat. Rev. Clin. Oncol..

[8] Ribas A., Sengupta R., Locke T., Zaidi S.K., Campbell K.M., Carethers J.M., Jaffee E.M., Wherry E.J., Soria J.C., D’Souza G. (2020). Priority COVID-19 vaccination for patients with cancer while vaccine supply is limited. Cancer Discov..

[9] Ong M.B.H. Cancer Groups Urge CDC to Prioritize Cancer Patients for COVID-19 Vaccination. https://cancerletter.com/articles/20210108_2/.

[10] SITC Statement on SARS-CoV-2 Vaccination and Cancer Immunotherapy. https://www.sitcancer.org/aboutsitc/press-releases/2020/sitc-statement-sars-cov-2-vaccination-cancer-immunotherapy.

[11] National Comprehensive Cancer Network Preliminary Recommendations of the NCCN COVID-19 Vaccination Advisory Committee* Version 1.0 1/22/2021. https://www.nccn.org/covid-19/pdf/COVID-19_Vaccination_Guidance_V1.0.pdf.

[12] Sociedad Española de Oncología Médica (SEOM) Posicionamiento y Recomendaciones de Seom en Relación con la Campaña de Vacunación Frente al COVID-19 en Pacientes Con Cáncer. https://seom.org/images/Posicionamiento_SEOM_vacunacion_COVID19_pacientes_con_cancer.pdf.

[13] Garassino M.C., Vyas M., de Vries E.G.E., Kanesvaran R., Giuliani R., Peters S. (2021). The ESMO Call to Action on COVID-19 vaccinations and patients with cancer: Vaccinate. Monitor. Educate. Ann. Oncol..

[14] AIOM CIPOMO COMU Documento AIOM CIPOMO COMU Vaccinazione COVID-19 per i Pazienti Oncologici ver 1.0. https://www.aiom.it/wp-content/uploads/2020/12/20201231_Vaccino_COVID_19_AIOM_CIPOMO_COMU_1.0.pdf.

[15] Polack F.P., Thomas S.J., Kitchin N., Absalon J., Gurtman A., Lockhart S., Perez J.L., Marc G.P., Moreira E.D., Zerbini C. (2020). Safety and efficacy of the BNT162b2 mRNA Covid-19 vaccine. N. Engl. J. Med..

[16] Baden L.R., El Sahly H.M., Essink B., Kotloff K., Frey S., Novak R., Diemert D., Spector S.A., Rouphael N., Creech C.B. (2021). Efficacy and safety of the mRNA-1273 SARS-CoV-2 vaccine. N. Engl. J. Med..

[17] Thakkar A., Gonzalez-Lugo J.D., Goradia N., Gali R., Shapiro L.C., Pradhan K., Rahman S., Kim S.Y., Ko B., Sica R.A. (2021). Seroconversion rates following COVID-19 vaccination among patients with cancer. Cancer Cell.

[18] Iacono D., Cerbone L., Palombi L., Cavalieri E., Sperduti I., Cocchiara R.A., Mariani B., Parisi G., Garfuli C. (2021). Serological response to COVID-19 vaccination in patients with cancer older than 80 years. J. Geriatr. Oncol..

[19] Monin L., Laing A.G., Muñoz-Ruiz M., McKenzie D.R., del Molino del Barrio I., Alaguthurai T., Domingo-vila C., Hayday T.S., Graham C., Seow J. (2021). Safety and immunogenicity of one versus two doses of the COVID-19 vaccine BNT162b2 for patients with cancer: Interim analysis of a prospective observational study. Lancet Oncol..

[20] Massarweh A., Eliakim-Raz N., Stemmer A., Levy-Barda A., Yust-Katz S., Zer A., Benouaich-Amiel A., Ben-Zvi H., Moskovits N., Brenner B. (2021). Evaluation of Seropositivity Following BNT162b2 Messenger RNA Vaccination for SARS-CoV-2 in Patients Undergoing Treatment for Cancer. JAMA Oncol..

[21] Pimpinelli F., Marchesi F., Piaggio G., Giannarelli D., Papa E., Falcucci P., Pontone M., Di Martino S., Laquintana V., La Malfa A. (2021). Fifth-week immunogenicity and safety of anti-SARS-CoV-2 BNT162b2 vaccine in patients with multiple myeloma and myeloproliferative malignancies on active treatment: Preliminary data from a single institution. J. Hematol. Oncol..

[22] Herishanu Y., Avivi I., Aharon A., Shefer G., Levi S., Bronstein Y., Morales M., Ziv T., Arbel Y.S., Scarfò L. (2021). Efficacy of the BNT162b2 mRNA COVID-19 vaccine in patients with chronic lymphocytic leukemia. Blood.

[23] Liberati A., Altman D.G., Tetzlaff J., Mulrow C., Gøtzsche P.C., Loannidis J.P.A., Clarke M., Devereaux P.J., Kleijnen J., Moher D. (2009). The PRISMA statement for reporting systematic reviews and meta-analyses of studies that evaluate healthcare interventions: Explanation and elaboration. BMJ.

[24] Sadoff J., Le Gars M., Shukarev G., Heerwegh D., Truyers C., de Groot A.M., Stoop J., Tete S., Van Damme W., Leroux-Roels I. (2021). Interim Results of a Phase 1-2a Trial of Ad26.COV2.S Covid-19 Vaccine. N. Engl. J. Med..

[25] Ueda M., Martins R., Hendrie P.C., McDonnell T., Crews J.R., Wong T.L., McCreery B., Jagels B., Crane A., Byrd D.R. (2020). Managing Cancer Care During the COVID-19 Pandemic: Agility and Collaboration Toward a Common Goal. J. Natl. Compr. Canc. Netw..

[26] Onder G., Rezza G., Brusaferro S. (2020). Case-Fatality Rate and Characteristics of Patients Dying in Relation to COVID-19 in Italy. JAMA.

[27] The Lancet Oncology (2021). COVID-19 and cancer: 1 year on. Lancet Oncol..

[28] US Centers for Disease Control and Prevention Interim Clinical Considerations for Use of COVID-19 Vaccines. https://www.cdc.gov/vaccines/covid-19/info-by-product/clinical-considerations.html.

